# Lung function and prevalence of respiratory symptoms in Norwegian crab processing workers

**DOI:** 10.1080/22423982.2017.1313513

**Published:** 2017-04-20

**Authors:** Marte R. Thomassen, Lisbeth Aasmoe, Berit E. Bang, Tonje Braaten

**Affiliations:** ^a^ Department of Occupational and Environmental Medicine, University Hospital North Norway, Tromsø, Norway; ^b^ Faculty of Health Sciences, Department of Community Medicine, UiT The Arctic University of Norway, Tromsø, Norway; ^c^ Faculty of Health Sciences, Department of Medical Biology, UiT The Arctic University of Norway, Tromsø, Norway

**Keywords:** Occupational asthma, respiratory health, crab processing, healthy worker effect, occupational exposure, crustaceans, king crab, edible crab

## Abstract

**Background**: Seafood processing workers have an increased risk of developing occupational asthma. This has not been studied among Norwegian crab processing workers, nor has the respiratory health of exposed workers been compared to a control group.

**Objectives**: Assessing the impact of working in the crab processing industry on workers’ respiratory health.

**Design**: A cross-sectional study of the respiratory health in two types of crab processing workers compared to a control group.

**Methods**: The study included 148 king crab (*Paralithodes c* *amtschaticus*) workers, 70 edible crab (*Cancer pagurus*) workers and 215 controls. Workers answered a questionnaire and performed spirometry measurements. χ^2^ and Fishers exact tests were performed on self-reported respiratory symptoms. Regression analyses and t-tests were used to assess lung function values.

**Results**: Self-reported respiratory symptoms were higher among crab processing workers compared to controls, and higher among king crab workers compared to edible crab workers. There was no significant difference between crab processing workers and controls in lung function measurements. Self-reported doctor-diagnosed asthma prevalence was highest in the control group.

**Conclusions**: Increased respiratory symptoms reported by crab processing workers were not reflected in impaired lung function values or asthma diagnose. We suggest a healthy worker effect among crab processing workers in Norway.

## Background

The seafood industry has played an important role in Norwegian history, and Norway is the largest fishery nation among the Nordic countries. The prevalence of occupational asthma in seafood processing workers has been estimated at 2–36% [1–4], with more frequent symptoms in workers who process crustaceans than in workers who process bony fish [2,5,6]. In the Canadian snow crab industry, occupational asthma has been found in approximately 16% of the workers [2,7,8]. A risk factor for developing occupational health problems is exposure to bioaerosols, which are generated during seafood processing. Bioaerosols from air samples collected in the seafood industry have been found to contain high-molecular-weight proteins, microorganisms, endotoxins and enzymes [9–14]. These bioaerosol components have previously been identified as risk factors for immunological sensitisation, respiratory symptoms, bronchial hyper-responsiveness and occupational asthma [1,5,7,8,15–19]. Variations between processing plants, such as building parameters and processing technologies, may contribute to differences in worker exposure levels [20]. Both edible crab (*Cancer pagurus*) and king crab (*Paralithodes camtschaticus*) are processed in land-based processing plants along the Norwegian coastline [12]. Edible crab is an indigenous species and has been part of the commercial fishing industry in Norway since 1914 [21]. The processing plant has a modern processing line and is optimised for crab processing, with separate areas for raw and cooked crab. Workers stand closely together and use automated brushes that rotate at a high speeds and conveyer belts to transport the crab.

The king crab is an invasive species that migrated from Russia to the coastal areas of North-Eastern Norway, and its commercial fishing started in 2002 [22]. Changes in stock migration and the regulation of capture quotas have resulted in crab processing often being side-lined for small, land-based fish processing plants. These plants are not built for crab processing; the crab processing equipment is placed in the plant during the crab processing season and is removed when the season is over. The processing lines are not optimally placed, especially in the cooking areas. Workers often take on different tasks during their shifts, including the handling of both raw and cooked crab.

Studies from snow crab production in Canada and Alaska have found that crab processing workers are at a high risk of developing occupational asthma [3,7,8,23–25]. Upper respiratory problems such as rhinitis and hay fever are risk factors for asthma and often precede the development of the disease [26–30]. Nonetheless, most studies in the seafood industry focus on diagnosing occupational asthma, and not on the presence of respiratory symptoms. We feel that self-report questionnaires assessing respiratory symptoms have great value for the early detection and implementation of measures to prevent the development of occupational asthma. Few studies have compared exposed seafood workers to a reference population that has not been occupationally exposed to crab processing. The objective of the present study was to assess the impact of working in the crab processing industry on respiratory health. We aim to accomplish this by comparing the lung function and the prevalence of respiratory symptoms of crab processing workers to a control group of workers who were never occupationally exposed to seafood.

## Methods

### Study design

The objective of the present study was to assess the impact of working in the crab processing industry. A cross-sectional study was done among workers on land-based facilities that process king crab (*P*. *camtschaticus*) or edible crab (*C*. *pagurus*) and among a control group of workers who had never worked in any kind of seafood industry. Control group data were collected between November 2007 and April 2008 and included municipal workers living in Norwegian coastal areas who had never worked in the seafood industry. Data from king crab processors were collected between autumn 2009 and autumn 2011, and from edible crab processors during autumn 2011. Companies registered as buyers of crab in the Norwegian Fishermen’s Sales Organization were invited to participate in a questionnaire study. Those businesses that agreed to participate received a questionnaire for each employee with a return envelope and a letter informing them about the study and its objectives. Employees from one edible crab plant and from five king crab plants participated in health examinations. All health examinations were performed at the workplace during normal working hours. There was no monetary compensation, but workers’ salaries were not decreased due to the time that they spent away from their work. Crab processing workers were taken out of production to perform the tests and returned after they had finished. The control group came at their convenience during their workday. To help with the questionnaire, contact information was given, and a researcher was available to provide clarifications.

Written informed consent was obtained from all participants for both the questionnaires and the health examinations. The study was approved by the Regional Committee for Medical Research Ethics.

### Study populations

The inclusion criteria for the exposed population were people working in a plant that processes either king crab or edible crab for at least 50% of their workday at the time of the study. The crab processing groups consisted of 154 king crab workers and 70 edible crab workers. For the health examinations, 139 king crab workers from five plants (91% of the eligible work force) and 70 edible crab workers from one processing plant (100% of the eligible work force) participated in one or more of the examinations. Due to unforeseen regulations, which ended the king crab fishing season during the collection of questionnaire data, very few plants that did not participate in the health examinations received the questionnaire while the plants were operating, so the response rate was only 23%. The control population consisted of people working in administrative organisations and schools in four coastal communities. The 215 employees included in the control group had never worked in the seafood industry.

### Questionnaire

The questionnaire used in the present study was a modified version of a questionnaire that was developed for previous Norwegian fishing industry studies [14,31] and included questions from the respiratory symptoms questionnaire recommended by the British Medical Research Council [32]. Responses were anonymous and were not made available to employers. Identical questionnaires were available in Norwegian and English and contained questions on age, gender, smoking habits, adult asthma, allergies and/or eczema in addition to respiratory symptoms. All questions regarding respiratory symptoms were limited to the past 12 months. The questions included the following: “Have you experienced wheezing in the last 12 months?”, and, if “yes”, “Did you also experience shortness of breath?”; “Do you usually cough or hem in the morning?”, and, if “yes”, “Do you usually have expectoration?”; “Do you cough daily/almost daily for, on average, 3 months or more throughout the year?”; and “In the last 12 months, did you experience a runny nose or nasal congestion that was not associated with a cold or the flu?”, and, if “yes”, “Did you have itchy, runny eyes at the same time?”

### Lung function measurements

To measure the subjects’ lung function, spirometry was performed using a Spida USB (CareFusion 234 GmbH, Hoechberg, Germany). The Spida USB is a stable instrument that does not require calibration, so it was not calibrated in the field. The same instrument was used for all measurements, and data were collected during the day shift at least 1 hour after the start of the shift. To calculate predicted lung function values, data were collected on gender, age and height. Workers were instructed not to smoke for 2 hours before testing, but no restrictions were made on the use of asthma medication. The forced expiratory volume in the first second of exhalation (FEV_1_) and forced vital capacity (FVC) were measured by instructing the person to expire forcefully after a full inspiratory manoeuvre. This was repeated until the test satisfied the American Thoracic Society 1995 criteria [33], but no more than 8 times. The highest values of FEV_1_ (l/s), FVC (l) and FEV_1_/FVC (%) were retained for analyses. Calculations of the predicted values were based on the equations proposed by Langhammer et al. [34] in a healthy, non-smoking, Norwegian adult population. Reduced lung function was classified by a FEV_1_ and/or FVC of less than 80% of predicted values. Airway obstruction was characterised as FEV_1_/FVC below the 5th percentile of the predicted values [35,36].

### Statistical analyses

Continuously measured variables were represented as the mean values with standard deviations, and categorical variables were represented as numbers with percentages. Crude analyses were performed using independent-samples t-tests, Pearson χ^2^ tests and Fisher exact tests. A multivariable linear regression model was applied for the analysis of continuous data from spirometric variables. Multivariable logistic regression analysis was used to test differences in dichotomous variables (FEV_1_ and FVC less than 80% of predicted, FEV_1_/FVC below the 5th percentile of predicted and respiratory symptoms). To improve efficiency, multiple imputation [37] was executed on all variables used in the regression models. The presented results of regression analyses are from the imputed dataset, whereas the descriptive results are from analyses on the non-imputed dataset. Statistical analyses were calculated using STATA 14, with p-values of <0.05 considered statistically significant.

## Results

### Characteristics of the study population

The study population consisted of 433 workers, of whom 212 were men and 221 were women. Ages ranged from 16 to 68 years, and total years of education ranged from 0 to 22 years. Compared to the control group, both king crab and edible crab workers were younger and had larger proportions of male workers (Table 1). Crab processing workers had lower levels of education and higher percentages of people who were smokers in their lifetime (former or current smokers). Prevalence of doctor-diagnosed asthma ranged from 3.2% in edible crab workers to 9.9% in king crab workers to 12.6% in controls. Differences in asthma prevalence were only significant between edible crab workers and controls. Allergy prevalence and family history of asthma, eczema and allergies were significantly higher in controls than in crab processing workers.

**Table 1.** Characteristics of the study population.

	Controls	King crab		Edible crab	
	n = 215	n = 148	p-value^a^	n = 70	p-value^a^
Age in years, mean (SD)^b^	47.4 (9.1)	38.7 (12.8)	<0.001	34.2 (10.4)	<0.001
Gender, n (%)					
Male	63 (29.3)	100 (67.6)	<0.001	49 (70.0)	<0.001
Female	152 (70.7)	48 (32.4)		21 (30.0)	
Smoking, n (%)					
Never	110 (51.4)	32 (22.4)	<0.001	22 (35.5)	0.027
Ever	104 (48.6)	111 (77.6)		40 (64.5)	
Pack-years, mean (SD)^b^	11.94 (14.8)	11.72 (11.2)	0.908	4.68 (5.9)	<0.001
Education in years, mean (SD)^b^	15.07 (2.9)	11.93 (2.5)	<0.001	12.95 (3.1)	<0.001
Asthma, n (%)	27 (12.6)	14 (9.9)	0.434	2 (3.2)	0.034
Eczema, n (%)^b^	37 (17.2)	14 (9.9)	0.052	0 (0.0)	<0.001
Allergy, n (%)	72 (33.5)	26 (18.3)	0.002	6 (9.7)	<0.001
Family asthma, n (%)^b^	82 (40.8)	46 (38.0)	0.622	6 (10.2)	<0.001
Family eczema, n (%)	83 (42.6)	23 (19.7)	<0.001	12 (20.7)	0.003
Family allergy, n (%)	73 (37.4)	15 (12.6)	<0.001	4 (6.9)	<0.001

*^a^* *p-values for independent-samples t-tests (continuous variables), Pearson χ^2^ tests (categorical variables) and Fisher exact tests (categorical variables with fewer than 5 observations in a group) between each of the exposed groups to the control group.*

*^a^* *Significant difference between king crab and edible crab workers.*

### Work-related respiratory symptoms and lung function

When stratifying by smoking (with ever-smokers consisting of former and current smokers and never-smokers consisting of workers who had never smoked), the prevalence of all respiratory symptoms was higher among ever-smoking workers than never-smoking workers in both the control and exposed groups (Table 2). Non-smoking crab processing workers reported a significantly higher prevalence than did non-smoking controls in all respiratory symptoms other than wheezing. There were no significant differences between crab processing workers and ever-smoking controls.

**Table 2.** Lung function values and respiratory symptoms among crab processing workers and controls stratified by smoking status.

	Controls		Exposed		p-value^a^	
	Never-smokers	Ever-smokers	Never-smokers	Ever-smokers	Never-smokers	Ever-smokers
Respiratory symptoms, n (%)	n = 108	n = 103	n = 44	n = 143		
Runny nose	29 (26.9)	30 (29.1)	15 (34.1)	46 (32.2)	0.621	0.725
Itchy/runny eyes^b^	17 (15.7)	15 (14.6)	9 (20.5)	18 (12.6)	0.930	0.200
Wheezing	8 (7.4)	16 (15.5)	6 (14.6)	34 (25.2)	0.177	0.070
Shortness of breath^b^	8 (7.4)	10 (9.7)	6 (14.6)	23 (17.0)	<0.001	0.720
Morning cough	14 (13.3)	20 (19.6)	12 (27.3)	43 (30.1)	0.041	0.065
Morning cough with phlegm^b^	3 (2.8)	15 (14.7)	9 (20.5)	22 (15.5)	0.016	0.090
Prolonged cough^c^	8 (7.6)	15 (15.3)	12 (31.6)	31 (22.5)	<0.001	0.171
Lung function values	n = 51	n = 59	n = 37	n = 101		
% of predicted FEV_1_ (l/s), mean % (SD)	90.8 (13.4)	90.7 (13.0)	92.5 (12.2)	90.1 (13.0)	0.559	0.782
% of predicted FVC (l), mean % (SD)	85.3 (12.9)	86.8 (13.2)	90.47 (11.8)	84.9 (13.9)	0.062	0.390
FEV_1_/FVC (%), mean (SD)	83 (1.0)	84 (1.0)	82 (7.0)	83 (9.0)	0.381	0.586
FEV_1_ below 80% of predicted, n (%)	6 (11.8)	13 (22.0)	5 (13.5)	21 (20.8)	0.807	0.853
FVC below 80% of predicted, n (%)	16 (31.4)	19 (32.2)	9 (25.7)	34 (36.9)	0.570	0.550
FEV_1_/FVC below 5th percentile of predicted value, n (%)	0	0	6 (16.2)	9 (9.0)	0.004	0.027

*^a^* *p-values for independent-samples t-tests (continuous variables), Pearson χ^2^ tests (categorical variables) and Fisher exact tests (categorical variables with fewer than 5 observations in a group) between exposed workers and controls.*

*^b^* *If they answered “yes” to the question above, did they also experience this?*

*^c^* *Daily cough lasting more than 3 of the last 12 months.*

The number of workers reporting respiratory symptoms was higher among crab processing workers than among controls for wheezing, shortness of breath and prolonged cough (Table 3). There was no difference between the groups in prevalence of phlegmy cough, and daily morning cough was borderline but not statistically significant.

**Table 3.** Respiratory symptoms among crab processing workers compared to controls.

	Odds ratio	95% confidence intervals	p-value^a^
Runny nose	0.59	0.21–1.64	0.311
Itchy/runny eyes^b^	0.38	0.04–3.66	0.404
Wheezing	2.71	1.35–5.41	0.011
Shortness of breath^b^	2.77	1.27–6.05	0.005
Morning cough	0.62	0.98–3.26	0.059
Morning cough with phlegm^b^	1.82	0.84–1.95	0.129
Prolonged cough^c^	2.97	1.49–5.92	0.002

*^a^* *Odds ratios, 95% confidence intervals and p-values of self-reported respiratory symptoms adjusted for age, gender, smoking (pack-years), education and family history of asthma and allergy. The control group formed the reference category.*

*^b^* *If they answered “yes” to the question above, did they also experience this?*

*^c^* *Daily cough lasting more than 3 of the last 12 months.*

There were no significant differences in lung function measurements between crab processing workers and controls when using regression analyses on imputed data (Table 4). When stratifying for smoking status using t-tests and χ^2^ tests on unimputed data (Table 2), crab processing workers had a higher prevalence of FEV_1_/FVC below the 5th percentile of predicted values compared to controls. Upon comparing king crab and edible crab workers (Table 5), the FVC was significantly lower in king crab workers. Additionally, more king crab workers had a FVC below 80% of predicted compared to edible crab workers. However, more edible crab workers had a significantly higher prevalence of FEV_1_/FVC below the 5th percentile of predicted values compared to king crab workers.

**Table 4.** Lung function values of crab processing workers compared to controls.

	β (95% confidence interval)	Odds ratio (95% confidence interval)	p-value^a^
FEV_1_ (l)^a^	−0.04 (−0.15 to 0.07)		0.485
FVC (l)^a^	0.01 (−0.13 to 0.16)		0.858
FEV_1_/FVC^a^	−0.01 (−0.02 to 0.01)		0.192
FEV_1_ <80% of predicted value^b^		1.07 (0.65–1.76)	0.788
FVC <80% of predicted value^b^		0.67 (0.42–1.06)	0.084
FEV_1_/FVC <5th percentile of predicted value^b^		1.24 (0.58–2.68)	0.578

*Regression coefficients (β) and 95% confidence intervals of spirometric variables (continuous scale) adjusted for age, gender, smoking, education and family history of asthma and allergy.

*^b^* *Odds ratios and 95% confidence intervals of reduced lung function (dichotomous scale) adjusted for age, gender, smoking, education and family history of asthma and allergy. The control group formed the reference category.*

**Table 5.** Respiratory symptoms and lung function values between king crab processing workers and edible crab processing workers.

	King crab	Edible crab	p-value^a^
Respiratory symptoms, n (%)	n = 138	n = 56	
Runny nose	48 (34.8)	9 (16.1)	0.036
Itchy/runny eyes^b^	21 (15.2)	2 (3.6)	0.718
Wheezing	30 (21.7)	10 (17.9)	0.500
Shortness of breath^b^	27 (20.3)	2 (3.6)	0.000
Morning cough	42 (30.4)	13 (23.2)	0.377
Morning cough with phlegm^b^	25 (18.1)	6 (10.7)	0.396
Prolonged cough^c^	32 (23.2)	11 (19.6)	0.592
Lung function values	n = 98	n = 52	
% of predicted FEV_1_ (l/s), mean % (SD)	89.7 (12.7)	93.9 (12.0)	0.092
% of predicted FVC (l), mean % (SD)	82.6 (13.3)	92.91 (10.1)	0.000
FEV_1_/FVC (%), mean (SD)	83 (9.0)	83 (7.7)	0.888
FEV_1_ below 80% of predicted, n (%)	20 (20.4)	6 (11.5)	0.233
FVC below 80% of predicted, n (%)	38 (38.8)	5 (9.6)	0.000
FEV_1_/FVC below 5th percentile of predicted value, n (%)	0 (0.0)	15 (28.9)	0.000

*^a^* *p-values for independent-samples t-tests (continuous variables), Pearson χ^2^ tests (categorical variables) and Fisher exact tests (categorical variables with fewer than 5 observations in a group) between king crab and edible crab workers.*

*^b^* *If they answered “yes” to the question above, did they also experience this?*

*^c^* *Daily cough lasting more than 3 of the last 12 months.*

## Discussion

In this study, lung function and prevalence of respiratory symptoms were measured among king crab and edible crab processing workers and were compared to a control population. The prevalence of some lower respiratory symptoms was significantly higher among crab processing workers than among controls. When stratifying for smoking, both never-smoking and ever-smoking crab processing workers reported a higher prevalence of respiratory symptoms than did their respective controls, but not all of these differences were significantly higher. There were no differences between crab processing workers and controls in lung function parameters. King crab workers had a significantly higher prevalence of shortness of breath than edible crab workers and a higher prevalence of reduced FVC measurements and FEV_1_/FVC below the 5th percentile of predicted values.

Prevalence of self-reported doctor-diagnosed asthma from the questionnaire was highest among controls. Asthma prevalence has been reported to be 8–10% in the general Norwegian adult population [38]. Whereas 12.6% of the control group reported having asthma, only 9.9% of the king crab workers and 3.2% of the edible crab workers reported having asthma. Previous studies on snow crab processing in Canada and Alaska show occupational asthma in approximately 16% of workers [2,7,8,20], with the levels varying between plants. A study by Cartier et al. reported prevalence rates of occupational asthma in different plants ranging from 9–50% [24]. Differences in prevalence between studies on occupational asthma may be due to differences in data collection or varying definitions of asthma. There may also be a “plant effect” [20], where plant layouts and characteristics such as building sizes, ventilation and shielding of work tasks affect exposure levels. The processing procedures and use of automated machines during processing influence the workers’ exposure and may affect the development of diseases such as asthma [11,20]. Changes in processing equipment, ventilation and personal protective equipment worn by the workers may also change over time. The edible crab industry used more automated equipment for processing, while the king crab industry used more manual procedures. The changes in processing techniques may have caused the differences between the populations in this study and could cause difficulties when comparing this study to previous studies in the crustacean industry. Since the king crab industry was a new industry during data collection, the methods for processing had been imported from Canada and were not developed in Norway, so the differences between the processing lines in the two countries should be small.

Self-reported wheezing, shortness of breath and prolonged cough were significantly more prevalent among crab processing workers compared to controls (Table 3). King crab workers also reported a significantly higher prevalence of runny nose and shortness of breath than did edible crab workers (Table 5). The edible crab processing plant used more automated processing, and measurements in the workers’ breathing zones showed higher levels of allergens in the edible crab plant compared to the king crab plants. Several studies on seafood processing workers have found a high prevalence of respiratory symptoms [7,39–41]. This supports the view that workers in crab processing industries are at increased risk of developing respiratory symptoms and may have more respiratory problems than non-exposed controls. When stratifying for smoking status, exposed workers still reported a higher prevalence of respiratory symptoms than controls (Table 2). The fact that non-smoking crab-processing workers reported more symptoms did than non-smoking controls suggests that crab processing is linked to the development of respiratory health problems. A study on king crab processing workers in Alaska reported wheezing in 37%, prolonged cough in 22% and morning phlegm in 25% of workers [42]. These percentages are similar to our findings among king crab workers and are higher than those of the edible crab workers in our study. Ortega et al. reported a 14% prevalence rate of asthma-like symptoms in Alaskan snow crab processing workers in the beginning of their study, and a 32% prevalence rate after 6 weeks of processing, with the symptoms being highest in the areas of butchering and de-gilling [39].

There was no difference in lung function parameters between crab processing workers and controls (Tables 2 and 4), apart from crab processing workers showing a significantly higher prevalence of FEV_1_/FVC below the 5th percentile of predicted values when stratifying by smoking status. This may indicate an obstructive lung disease, such as asthma or chronic obstructive pulmonary disease (COPD), in the exposed crab processing workers. To verify the presence of asthma, the workers must demonstrate an obstruction with reversibility via repeated testing, or that the obstruction could be provoked by inhalation testing. Since significant differences were not found in the regression analyses in which we adjusted for smoking, the difference in lung function may also have been due to COPD and could have been caused by the significantly higher prevalence of workers who smoked in the exposed group.

When comparing king crab and edible crab workers, the king crab workers had significantly lower percentiles of predicted FVC and higher prevalence rates of workers with reduced FVC, but no difference was found in FEV_1_. This may indicate a restrictive lung problem that is not necessarily linked to an illness in the lungs, but may be caused by other factors, such as a large stomach making it difficult for the subject to inhale properly. Edible crab workers had significantly more workers with FEV_1_/FVC below the 5th percentile of predicted value compared to king crab workers. This may indicate an obstructive lung function and possibly asthma. To confirm whether the findings indicate asthma, further testing needs to be done.

The results of the spirometry measurements may be an underestimation of the asthma prevalence among crab processing workers. All spirometry measurements were taken during work shifts, when the workers were exposed to the crabs. This would capture workers who had an immediate reaction to the work conditions, but not those who developed a late asthmatic reaction [7]. Ortega et al. found similar results in a population of snow crab processing workers where the workers reported respiratory symptoms that were not reflected in the spirometric measurements [39]. Our measurements were taken during the day shift after workers had been at work for at least 1 hour, so diurnal effects should be low. However, measurements were taken on two consecutive days, so if workers demonstrated a tolerance, which has been found in previous studies [8,19,42], the day on which the measurements were taken could have affected the spirometry results. The workers had not been instructed to avoid taking asthma medication before the examination, which may also have led to an underestimation of reduced lung capacity. However, due to the small number of crab processing workers with asthma (16 workers), crab processing workers were not instructed to avoid taking asthma medications, as the number was so low it was unlikely to have impacted the results.

Workers who already have asthma or develop severe respiratory problems or asthma may tend to either avoid working in crab processing plants or discontinue this type of work. As a result, the disease levels among crab processing workers may be underestimated because the study did not include those who left the crab processing industry because of illness. This is an example of a well-known selection bias in cross-sectional occupational studies that may mask the harmful effect of an occupational environment [43–45]. This “healthy worker effect” may explain the low prevalence of asthma and allergies in our study compared to others. In communicating with the workers and leaders at the processing plants, it was well known that some workers chose not to return to the plant due to the health problems that they developed at work. In Norway, there are systems that provide financial aid for people who cannot work, so the threshold to leave work may be lower than those of other countries. The lower prevalence of family asthma, allergy and eczema may also explain some of the difference, because allergic diseases are known to have a genetic predisposition [46]. Early intervention is important, as the prognosis for recovery depends on the duration of exposure after symptoms occur [47]. If workers are diagnosed early and removed from exposure, their health may improve. However, once they are sensitised to an allergen, the condition is typically not reversed. Hudson et al. discovered that improvement plateaued 2 years after removal from work, which emphasises the importance of the early discovery of symptoms [47]. Thus, an early response to symptoms reported by workers can prevent the development of occupational disease by implementing protective measures such as ventilation, shielding of work tasks or fitting workers with personal protective equipment.

Our study has some limitations. Due to the small sample size, the differences between crab processing workers and controls may not be visible due to a lack of power. To adjust for this, we used multiple imputations for missing observations in order to perform the analyses on the complete dataset. This does not alter the effect estimates of the outcome, but increases the precision and the power of our findings. There were also significant differences in age, gender, smoking, education and disease history. To adjust for this, these variables were included in the regression analyses. It would also have been relevant to adjust for existing asthma and allergies among the workers, but due to the cross-sectional design of the study, separating work-related asthma from pre-existing asthma was difficult, and such adjustments may have led to an over-adjustment of the association between the predictive variables and the outcome. A questionnaire was used to find the prevalence of doctor-diagnosed asthma and respiratory symptoms. This may have caused an underestimation of asthma prevalence if the workers had not visited their doctors and obtained a diagnosis of asthma prior to the study. The subjects were asked to retrospectively fill in details on any respiratory symptoms they had experienced in the last 12 months, which may have been affected by recall bias. Because most workers completed the questionnaire, we believe that the results are representative of the crab processing plants and that there was no selection bias where only those demonstrating health problems participated.

## Conclusions

This study demonstrates that working in king crab and edible crab processing plants is associated with greater levels of respiratory symptoms compared to a healthy control population. The respiratory symptoms were not reflected by impaired lung function values or increased asthma diagnosis. Based on a lower prevalence of asthma and allergies and a higher prevalence of respiratory symptoms among crab processing workers compared to controls, we suggest that there is a healthy worker effect among crab processing workers. Further studies are needed in order to better understand the development of respiratory health problems and potential risk factors affecting these workers’ health problems. With a better understanding of the risks, it would be possible to implement preventative measures. A cohort study of new employees in crab processing plants linking respiratory outcomes with exposure measurements taken during processing could be useful in helping to map the incidence of occupational health problems and to identify possible causal factors in the work environment.
